# The Potential of Wetlands as Treatment Systems for Organic Matter and Some Selected Metals (As, Ca, Cd, Cr, Cu, Fe, K, Mg, Mn, Na, Pb, and Zn): Case Study of Yitamot Wetland, Ethiopia

**DOI:** 10.1155/2022/3824883

**Published:** 2022-06-07

**Authors:** Yezbie Kassa, Seyoum Mengistu, Ayalew Wondie, Dessie Tibebe

**Affiliations:** ^1^Department of Biology, College of Natural and Computational Sciences, University of Gondar, P. O. Box 196, Gondar, Ethiopia; ^2^Department of Zoology, College of Natural and Computational Sciences, Addis Ababa University, P. O. Box 176, Addis Ababa, Ethiopia; ^3^Department of Biology, College of Natural and Computational Sciences, Bahir Dar University, P. O. Box 96, Bahir Dar, Ethiopia; ^4^Department of Chemistry, College of Natural and Computational Sciences, University of Gondar, P. O. Box 196, Gondar, Ethiopia

## Abstract

Organic matter and some selected metals (As, Ca, Cd, Cr, Cu, Fe, K, Mg, Mn, Na, Pb, and Zn) were measured from water and sediment while plant samples from the inlet to the outlet sampling sites using standard procedures from three compartments (water, sediment, and macrophytes) of Yitamot wetland. Findings indicated that the mean concentration of COD in water was in the range of 5.25 mg/L and 208.25 mg/L and showed a significant and exponential decrease along the subsequent sampling sites (*P* < 0.05). The mean concentrations of K, As, Cd, and Cr (82.192 mg/kg, 0.730 mg/kg, 0.06 mg/kg, and 19.776 mg/kg, respectively) were significantly lower values in the outlet than in the inlet of the sediment samples (*P* < 0.05). All the metal concentrations in the aboveground tissue of macrophytes were significantly lower at the outlet sample site (*P* < 0.05) indicating that these compartments are contributing to the retention of metals and organic matter present in wastewater discharged in the wetland through sinks and conversions of these compounds. However, there was no significant removal effect for heavy metals like Fe, Zn, and As (*P* > 0.05). This is a pointer to the fact that natural wetlands can be used effectively for wastewater treatment with strong monitoring programs and ecological indicators for the sustainable management and conservation of the flora and fauna present in it.

## 1. Introduction

Pollutant removal by wetlands is highly dependent on hydraulic loading and influent concentration and to a lesser extent on internal plant communities, water depth, and hydraulic efficiency. Aquatic plants are suitable for wastewater treatment with a high capacity of removing nutrients and other substances through uptake, sorption, and microbiological degradation ([1]).

In most countries of the world, partly treated and sometimes untreated wastewater with nutrients is discharged directly to surface waters or through wetlands which are in close proximity to urban centers [2, 3]. Africa, in particular Ethiopia, is no exception to this problem [4]. The presence of these wastes in water bodies becomes undesirable, as it hinders the sustainable life of organisms ([5], [6]). Many Ethiopian natural wetlands are depleted and are under severe stress [4, 7]. There are also some research gaps regarding the sustainable utilization and conservation of Ethiopian wetlands. The expansion of human settlement and industrialization around wetlands in some parts of the country, e.g., Bahir Dar, Hawassa, and Debrezeit, has become an important source of pollution and is threatening the stability of these ecosystems [4]. Other major threats include deforestation, over grazing, planting of high-water demanding plants, spread of invasive plants species, drainage for agriculture, siltation, pesticides and fertilizer applications in nearby farms, and construction of dams [7–9]. For instance, wetlands associated with Lake Tana recession agriculture and abstraction of water for small-scale irrigation during the dry season in Shesher and Megech wetlands and invasive alien species such as water hyacinth are the main threats identified in the study of [7]. The Megech, Gudo Bahir, and Shesher wetlands were seriously impaired by human activities (carried out within and their catchments) and hence had classified as high impaired wetlands [10]. The other wetlands such as Ras Abbay, Chimba, and Avaji were clustered as midimpaired wetland [11]. Moreover, as documented in many papers (e.g., [11–14]), human factors are the major contributors for wetland degradation and alteration of biological community composition. The threats facing the wetlands in the Main Ethiopian Rift show that the bases of the degradation of the wetlands are indeed human activities [15].

Yitamot wetland is located at the vicinity of Bahir Dar and is threatened with the increased inflow of nutrients and extensive encroachment for various land use activities, like agriculture, and high settlement densities and being an effluent discharge site [10]. The wetland is under threat of two main channels: the storm water drainage network direct water from the runoff especially during rainy seasons and untreated sanitary university effluents directly into the wetland. This system receives pulses of pollutants each time it rains, although it is believed that the wetlands help to prevent much of the pollutant load from being transported elsewhere (Abay/Blue Nile River in this case). The wetland received untreated effluents from the university continuously both dry and wet season, throughout the year. There is a possibility that pollutants (N and P) reach the Blue Nile River and can cause pollution of the river. Wetlands should be created or intervened for their sustainable management. As a result, information on water quality in the wetland is needed for the planning of sustainable use of the wetland. It is currently assumed that the Yitamot wetland retains the organic matter and heavy metals carried with the wastewater, but there is no quantification of this function. Such information is necessary for the efficient planning of long-term sustainable use of the wetland and its biodiversity. This study was carried out to address the uncertainty regarding the functioning of the Yitamot wetland and the lack of information on the processes taking place therein.

Therefore, this study was aimed at (1) evaluating the potential of the existing Yitamot wetland to remove organic matter and other pollutants like metals from wastewater discharged into it in a sustainable way, (2) describing conversions and sinks of organic matter and metals, and (3) suggesting options for sustainable management of the wetland, including the potential of the wetland to be used in a more optimal way for wastewater treatment while maintaining ecological and biodiversity quality.

## 2. Materials and Methods

### 2.1. Study Site Description

**Figure 1:**
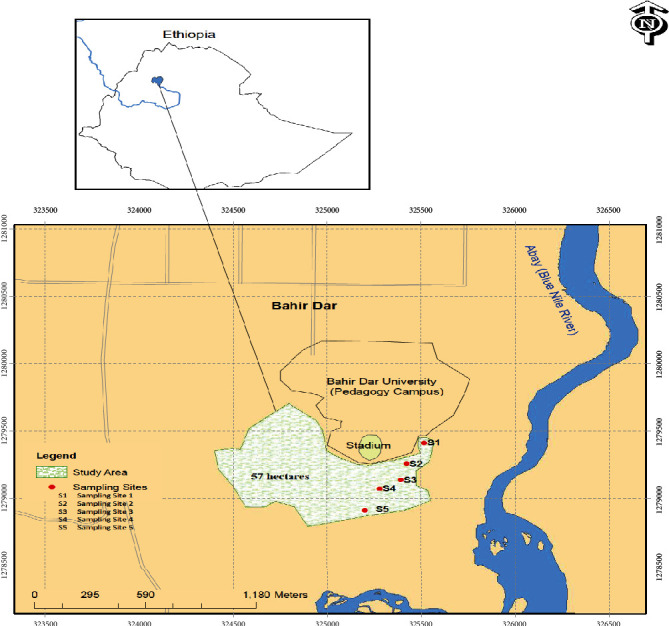
Map of Yitamot wetland indicating the sampling sites.

Yitamot wetland is a wetland located at 37°23′51.7^″^ of East and 11°33′58.2^″^ of North with an altitude of 1790 meter above sea level. It is located in Amhara National Regional State in the city of Bahir Dar, in the southern side of Bahir Dar University Pedagogical Campus. The wetland is connected to Abay (Blue Nile) river in the east. It has an area of about 57 hectares (Figure 1). According to the Ethiopian agroecological zonation, the wetland and its surrounding areas lie in “Woina Dega” or subtropical zone, and the mean maximum temperature ranges from 24.17°C to 30.29°C and the mean minimum temperature ranges from 9.96°C to 14.51°C [16]. The maximum temperature usually occurs from March to May with the highest in April and the minimum temperature from November to February at its lowest during January [16].

The wetland is seasonal type and its size and water level vary at different seasons. The other limnological characteristics of the wetland also varied; for example, according to the study of Yezbie [16], pH is in range between 5.7 and 8.5, EC ranged from 553 *μ*s/cm to 1195 *μ*s/cm, DO ranged from 1.1 mg/L to 5 mg/L, and turbidity ranged from 10.8 to 276.4 NTU. It is predominantly fed by groundwater and/or spring flow, as well as surface flow during the wet season. According to the Ethiopian Meteorology Agency, Bahir Dar area has mean annual rain fall which varies between 628.30 and 3152.80 mm with a mean value of 1355.74 mm, where most of the rain occurs in July followed by August [16]. Generally being located in the same area with Lake Tana, the wetland has climatologically similar conditions.

The wetland has different emergent, floating, and submerged macrophytes with dominant species being Echinochloa stagnina, Cyperus papyrus, Phragmites karka, Sphaeranthus suaveolens, Cyperus digitatus, Scirpus validus, Typha latifolia, Ludwigia abyssinica, Polygonum senegalense and others. Floating-leaved species Hydrocotyle ranunculoides, Azolla africana, Pistia stratiotes, Nymphaea caerulea, and Nymphaea lotus and submergent Ceratophyllum demersum are noted as the most important plants with varied coverage seasonally [16]. Farmers living near the wetland have livelihoods directly linked to the wetland. They benefit from the wetland in several ways: for instance, a large majority of them are engaged in livestock grazing, and others are engaged in small-scale irrigation using water from the wetland mainly when it has enough water level. Apart from the economic benefit of the wetlands, people use water from the wetland for sanitation purposes and for household purposes, except drinking. All of the cattle, horses, and other animals from different parts of the city and surrounding area are entirely dependent on the wetland for drinking, in addition to grazing.

Anthropogenic activities are directly and indirectly generating a drastic change in the ecosystems of Yitamot wetland. For instance, besides agricultural runoff, every day activities such as excavation at construction site, driving, maintaining vehicles, disposing of wastes, and collecting wastes in failed septic systems and sewer structures contribute substantial amount of contaminant to runoff. The ever-increasing unwise utilization of resource coupled with land use and climate changes and agricultural expansion with its close proximity have become sever threats to both the long-term survival of Yitamot wetland and to prevent much of the pollutant load from being transported to the receiving water body (Abay/Blue Nile River).

### 2.2. Sampling Design

The study was designed to investigate the ability of Yitamot wetland to remove organic matter and some selected metal pollutants by analyzing chemical oxygen demand (COD) in the water and metals in the water, sediments, and dominant macrophytes using a series of five sampling points running in a transect along the longitudinal axis of the wetland from the inlet to the outlet at 100-meter interval (Figure 1).

To avoid distortion of the results by pulses of rainfall, which can greatly but temporarily increase pollutant loads, collection was made of surface water, sediments, and macrophyte samples in dry season months (January and May) twice a month and averaged. During each sampling time, triplicate measurements were done. The parameter COD was analyzed in water, and twelve metals (As, Ca, Cd, Cr, Cu, Fe, K, Mg, Mn, Na, Pb, and Zn) were analyzed in three compartments (water, sediment, and plant) of the wetland.

#### 2.2.1. Organic Matter Determination from Water Sample

Water samples were collected from each sampling site to analyze for COD from unfiltered samples. The analytical method used for determination of COD was dichromate test method [17]. The dichromate chemical oxygen demand (COD) test measures the oxygen equivalent of the amount of organic matter oxidizable by potassium dichromate in a 50% sulfuric acid solution. Silver sulfate was used as a catalyst and mercuric sulfate was added to remove chloride interference.

In the process, water samples were added to a cuvette (with reagent) and left it in a heater (Model HACH DRB 200) for 2 hours. After the oxidation step was completed, the amount of dichromate consumed was determined colorimetrically. The intensity of color in the solution was directly related to the COD value in the sample and was measured with ultraviolet spectrophotometer (model HACH DR 2800) in Chinese Academy of Sciences (Research Center for Eco-Environmental Sciences (RCEES), Department of Water Pollution Control Technology laboratory).

#### 2.2.2. Metal Analysis in Water, Sediment, and Plant Tissue

Water, sediment, and plant tissue samples were collected from each sampling site and analyzed for metals (As, Ca, Cd, Cr, Cu, Fe, K, Mg, Mn, Na, Pb, and Zn) concentration following the procedures outlined in US EPA [18], Method 200.7 d.

The composite water sampling method was used for collection of water sample in each sampling station twice covering a period of May 2014 and January 2015. 100 mL sample was collected in thoroughly cleaned polyethylene container bottles. The samples were preserved by adding 5 drops of nitric acid and stored below 4°C in a refrigerator before analysis. Sediment samples were taken from each study site for analysis of the above mentioned metals using a modified sediment core sampler according to Powel [19] from approximately 10 cm of the sediment surface. The collected sediment samples were thoroughly dried and pooled and ground to pass in a 100-mesh sieve (0.149 mm).

Plant samples were collected from five sampling sites along a transect from the inlet of effluent into the wetland to the outlet: S1, S2, S3, S4, and S5 (Figure 1) for plant tissue metal analysis. The biomass of macrophytes was obtained by using quadrat method (size of 0.25 m^2^ area) and harvesting the plants aboveground part as in Lee (1990). Two quadrats were laid in opposite side of each sampling site following Piotrowicz et al. (2006) and the quadrat samples were averaged. All aboveground material was placed in plastic bags and taken to the limnology laboratory of Addis Ababa University for determination of dry weight following the procedures in Wetzel and Likens (2000). From the dry weight of the biomass of each plant aboveground tissue, a representative sample was pooled and ground to pass a 100-mesh sieve.

Plant samples were also collected in all the sampling sites and rinsed *in situ*, blotted, pressed, and transported to the National Herbarium, Addis Ababa University, Ethiopia, for identification, which was made to the species level using Ethiopian flora such as those of [20, 21], and by matching with collections in the National Herbarium.

The preserved water samples were dried and pooled, and ground sediment and plant tissue samples were digested with a mixture of concentrated HCl-HNO_3_-HF-HClO_4_ in a microwave digestion system (MARS 230/60 CEM Mars Xtraction Model 907500) following the procedures in Bettinelli et al. [22] as follows.

In the process of digestion, 8 mL water sample was taken and 8 mL of HNO_3_ was added and taken to the microwave digestion (CEM) adjusted according to the US EPA [18], Method 200.7 d. After completion of digestion, ultrapure water was added to a final volume to 25 mL.

In the process of sediment digestion, 0.1 g grounded sediment samples were placed into digestion tank, 6 mL aqua regia (3 mL HCl: 1 mL HNO_3_) and 2 mL hydrofluoric acid were added, and the tank was taken to the microwave digestion (CEM) adjusted according to the EPA standard for digestion process. After digestion is completed, 1 drop of perchloric acid was added to catch the acid and the digestion tank was removed. Deionized water was added to the digested solution to a final volume of 10 mL.

For plant tissue digestion, 0.1 g plant tissue sample was pulverized using liquid nitrogen (100 mesh), 8 mL nitric acid was added to the sample and left overnight under Mars digestion (CEM recommended method), and in the next day (preferably after standing pure class), 2 mL 30% hydrogen peroxide (superior grade pure) was added to the plant samples.

The measurements of concentration in all the digestion solutions were performed using Inductively Coupled Plasma Optical Emission Spectrometer, PerkinElmer® Optima® 8300 ICP-OES coupled with the prep FAST™ Automated In-Line Auto-Dilution/Calibration System (Elemental Scientific Inc., Omaha, NE) in Chinese Academy of Sciences (Research Center for Eco-Environmental sciences (RCEES), Department of Water Pollution Control Technology laboratory).

#### 2.2.3. Pollutant Removal Efficiency of Yitamot Wetland

The pollutant removal efficiency (PRE) of the wetland was evaluated with respect to the pollutants like organic matter (COD) and metal elements in both influent (S1) and effluent (S5) concentrations during the monitoring period. The information collected from the analysis was used for computation of the pollutant removal efficiencies. PRE was calculated as percentage by the following equation:

(1) $$\begin{array}{c} \text{PRE}\%=\frac{C_{i}–C_{f}}{C_{i}}*100, \end{array}$$

where *C*_*i*_ is the influent concentration and *C*_*f*_ is the effluent concentration.

### 2.3. Statistical Analysis

Statistical analysis of results was done using Statistical Package for Social Sciences (SPSS) software package version 20. The significance of differences in variations in physicochemical variables and metal concentrations within the study sites and macrophyte species of the wetland were assessed statistically using analysis of variance (ANOVA) and Tukey's multiple comparisons for differences between means. In all tests, differences were considered statistically significant at *P* < 0.05.

## 3. Results

### 3.1. Organic Matter in Water Sample

The results of analysis obtained along the transect from the inlet to the outlet (S1, S2, S3, S4, and S5) are summarized in Table 1. More variation in mean values for COD was observed among the sites. The mean concentrations of COD were in the range of 5.25 mg/L to 208.25 mg/L and showed significant and exponential decrease along the subsequent sampling sites (*P* < 0.05).

### 3.2. Metal Concentration in Water Sample

**Figure 2:**
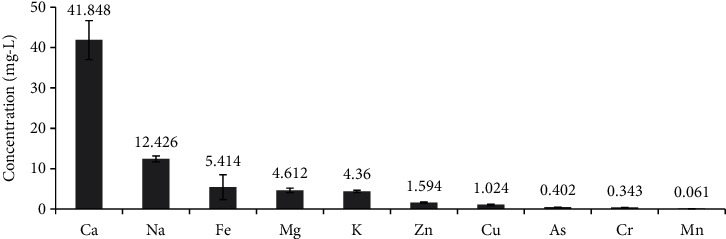
Means of metal concentration in Yitamot wetland water. Error bars indicate standard error of mean.

The mean concentrations of metals in the wetland water sample are shown in Figure 2 and the related information is summarized in Table 1. Based on the mean concentrations, the target elements in the water of Yitamot wetland exhibited the following descending order: Ca > Na > Fe > Mg > K > Zn > Cu > As > Cr > Mn, and the mean concentrations of Pb and Cd were not detected in all sampling sites.

The mean concentrations of metals were variable and some of them did not show significant variation among sampling sites (*P* > 0.05); for example, the mean concentrations of Ca and Mg were increased at the outlet (S5), but the variation was nonsignificant. However, the mean concentrations of Fe, Mn, and Zn in S3 were significantly higher than the other sites (*P* < 0.05), and the mean concentration of Mn was significantly lower at the outlet sampling site.

### 3.3. Metal Concentration in the Sediment

The mean concentrations of metals and related information in the sediment of the wetland are summarized in Table 2. Based on the mean concentrations, the target elements in the surface sediments of the Yitamot wetland exhibited the following descending order: Fe > K > Na > Ca > Mg > Mn > Cr > Zn > Cu > As > Pb > Cd.

The results of statistical analysis showed the mean concentrations of K, As, Cd, and Cr were significantly lower values in the outlet than in the inlet (*P* < 0.05). However, the mean concentrations of Fe and Pb were significantly higher in the outlet than the inlet sampling sites (*P* < 0.05), whereas the mean concentrations of Ca, Na, Mn, and Zn were significantly varied at the middle sampling sites but did not show significant variation between the inlet and the outlet sampling sites (*P* > 0.05). The mean concentrations of Cu and Mg did not show any significant variation among sites.

### 3.4. Metal Concentration in Plant Tissue

The results of this study for the mean metal concentration in each macrophyte species studied are presented in Table 3. The concentration of K, Fe, Cu, Zn, Cr, and As ranged from 75.87 mg/kg to 1159.33 mg/kg, 2.41 mg/kg to 213.03 mg/kg, 0.08 mg/kg to 0.85 mg/kg, 0.30 mg/kg to 4.22 mg/kg, 0.04 mg/kg to 0.81 mg/kg, and 0.11 mg/kg to 0.94 mg/kg, respectively. Na and Mn ranged from 4.16 to 299.62 mg/kg and 2.91 to 7.44 mg/kg, respectively, where the highest concentration was observed in *Sphaeranthus suaveolens* and the lowest concentration was observed in *Phragmites karka*. The concentration of Ca ranges from 15.29 to 122.49 mg/kg where the highest concentration was observed in *Hydrocotyle ranunculoides* and the lowest concentration was observed in *Cyperus papyrus*, and Mg in the plant tissue ranged between 2.48 and 30.33 mg/kg where the highest concentration was observed in *Echinochloa stagnina* and the lowest concentration was observed in *Cyperus papyrus.*

Each of the macrophytes has varied tendency of accumulation different metals. Most of the metals are in a highest concentration in *Sphaeranthus suaveolens* (Table 3). The highest accumulated metals in the tissue of all macrophytes studied were K, Na, Ca, Mg, and Fe in a consecutive order. Mn, Cu, Zn, Cr, and As were found in a small concentration. However, the concentrations of Pb and Cd in each species were very low and not detected.

**Figure 3:**
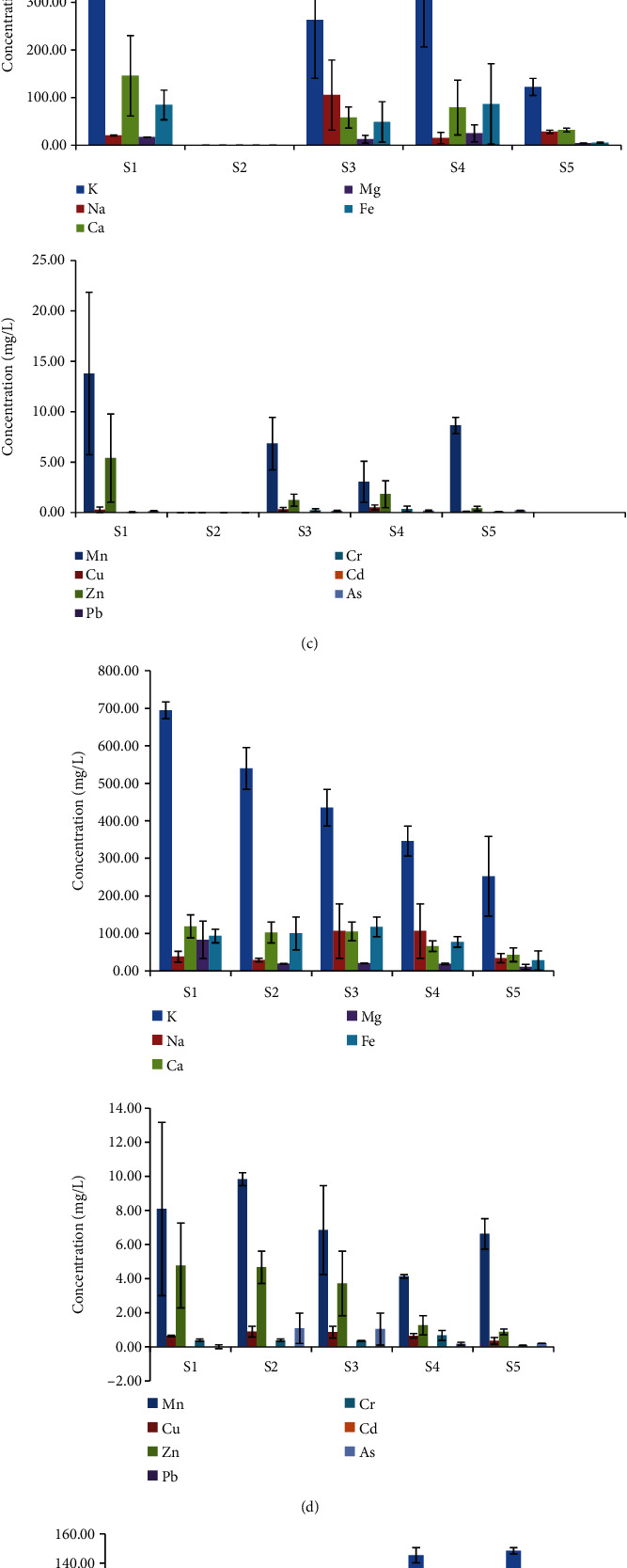
(a) Metal concentration (mg/L) in *Sphaeranthus suaveolens* aboveground tissue along sampling sites. (b) Metal concentration (mg/L) in *Hydrocotyle ranunculoides* aboveground tissue along sampling sites. (c) Metal concentration (mg/L) in *Cyperus digitatus* aboveground tissue along sampling sites. (d) Metal concentration (mg/L) in *Echinochloa stagnina* plant tissue along sampling sites. (e) Metal concentration (mg/L) in *Cyperus papyrus* aboveground tissue along sampling sites. (f) Metal concentration (mg/L) in *Phragmites karka* aboveground tissue along sampling sites.

The mean concentrations of metals in each species along sampling sites from the inlet to the outlet did not show regular trend (Figures 3(a)–3(f)). Statistical test and Tukey's multiple comparisons showed that the mean concentrations of K, Na, Fe, Mn, Cu, Zn, As, and Cr were significantly higher (*P* < 0.05) in aboveground tissue of *Sphaeranthus suaveolens* growing in S2 and sampling S3. In the same vein, concentrations of K, Na, Mg, As, and Zn showed a significant increase in aboveground tissue of *Hydrocotyle ranunculoides* growing in S2 and S3. However, in all the species studied, all the metal concentrations in the aboveground tissue of macrophytes were significantly (*P* < 0.05) lower at the last sample site from the inlet to the outlet where the species is found indicating that these macrophytes are contributing for the retention of metals in the wetland.

### 3.5. Organic Matter and Metal Removal Efficiency of Yitamot Wetland

The organic matter removal efficiency (PRE) of Yitamot wetland was evaluated with respect to the organic matter (COD), and metal elements in both influent and effluent concentrations in water were calculated and expressed as percentage as shown in Table 4.

Significant removal effect was observed in most pollutants like COD and metals like Mn, Cr, and Cu (>50%). The wetland showed the most removal effect (>97%) on COD and metals like Mn (>99.99). Negative percentages of removal were observed on metals like Mg (-40.41%) and Ca (-14%).

## 4. Discussion

### 4.1. Organic Matter and Metal Concentration in Water Column

The influent water (S1) and sampling S2 had COD above the Ethiopian effluent allowable discharge limits (100 mg/ L) into water courses [23]. The high concentrations of COD above the standard limit at S1 and S2 indicate a heavy load of organic and inorganic pollution that requires more oxygen to oxidize under increased thermal conditions. The mean value of COD in this study, although it was greater in the inlet sampling site than the Ethiopian effluent allowable discharge limits (100 mg/L) into water courses, showed a significant and exponential decrease along the subsequent sampling sites of (S2, S3, S4, and S5) and at the outlet the value fell under the limit mentioned above, indicating that the wetland and its components contributed to water quality improvement. The significant reduction in COD at the outlet can be attributed to biodegradation of the organic matter by microorganisms in the wetland. Vegetation in wetlands has a number of benefits that has a positive impact on most biological processes. The growth and development of roots and corms ensure the development of microorganisms, with a positive impact on microbiological processes [25]. The trapping of particulate organic matter by the wetland vegetation might have also contributed to decrease in COD at the outlet as the organic matter settle as sediment off the water column.

Pb and Cd were not detected in all sampling sites in this study indicating that wastewater receive little or no effluents contaminated with these metals. The mean concentrations of Ca, Mg, Fe, Mn, and Cr in the outlet (S5) of the wetland were under the Ethiopian effluent allowable discharge limits into water courses (150 mg/L, 150 mg/L, 10 mg/L, 5 mg/L, and 1 mg/L, respectively) [23]. On the other hand, the mean concentrations of Cu, Zn, and As though decreased at the outlet, in all sampling sites, were higher than the limits (0.1 mg/L, 1.0 mg/L, and 0.1 mg/L, respectively) [23]. And the mean concentrations of As, Cr, Cu, and Fe were also higher than the maximum allowable concentration ranges for fisheries and aquatic life set by [24] (0.05 mg/L, 0.02 to 0.002 mg/L, 0.002 to 0.004 mg/L, and 0.3 mg/L, respectively) which shows that Yitamot wetland can be regarded as relatively influenced by anthropogenic pollution. Heavy metals such as cadmium, chromium, copper, iron, nickel, lead, and zinc exhibit aquatic toxicity when present above recommended standard in that they can contaminate surface and groundwater bodies, soil, plant, aquatic life, and man, through bioaccumulation [26].

In view of the fact that the major use of water in the study area is for cattle drinking, domestic, and irrigation, the concentration levels of heavy metals Cu, Zn, and As recorded in this study exceeded the limit for aquatic ecosystem set by [24]; therefore, it is of great concern since these metals are extremely toxic and the consumption of water with high concentration of these metals could cause adverse health effect to end users. The high concentration of Cu, Zn, As, and Zn in the study area could be attributed to the discharge of domestic wastewater containing compounds of these metals. The domestic wastewater discharged into the wetland could be composed of grey water that may consist of the bath, dishwasher products, personal care products, and laundry detergents, which are good sources of these metal elements [27, 28]. Also, dumping of wood treated with chemicals made from salts of these metals (As, Cr, Cu, and Zn) to prevent fungi and pest attack might provide a potential source of chemical spills and drainage from the treated wood around the wetland; this is in support of other findings [29]. The presence of iron in the study area could be related to run-off from rusted metallic pipes at the scrap metal dump sites.

Variable mean concentrations of metals among sampling sites were also recorded; for example, the increased mean concentrations of Ca and Mg at the outlet sampling site (S5) though nonsignificant and the significantly high mean concentrations of Fe Mn and Zn in S3 than the inlet sampling site could be attributed to the chemical cycling of the metals. Some metals can be existed being binding to some other chemicals at the inlet (S1) but might be detected later because of certain chemical reactions; the bond may break and the metals can be detected at another sampling site (S3). For example, the high level of Fe recorded at S3 within the study area could be attributed to the higher temperature with increasing pH and low dissolved oxygen content at the site, thus increased microbial decomposition of organic particulate matter; consequently, iron can easily be released from the particulate organic matter in the environment. The case is also indicated in other studies [30]. The inclination of the wetland towards this sampling site leads the wastewater to accumulate more in the site than the other sampling sites. Also, high levels of copper above the limits could be due to the effluent containing copper metal chips from metal engineering operations involving Cu scrap. Although copper toxicity in humans is rare, aquatic organisms are potentially at risk from Cu exposures [31].

### 4.2. Sediment and Plant Tissue Metal Concentrations

With the combined action of adsorption, hydrolysis, and coprecipitation, only a small part of free metal ions stay dissolved in water, and a large quantity of them get deposited in the sediments [32]. As a result, the measurement of metals only in the water is not conclusive due to water discharge fluctuations and low resident time [33, 34]. The metal concentrations obtained from the sediment samples in this study were compared with US EPA Sediment Quality Guideline [35], and mean concentrations of Cd, Cr, Cu, and Zn exceed the probable effect concentration (PEC) which is 3.53 mg/kg, 90 mg/kg, 197 mg/kg, and 315 mg/kg, respectively. The mean concentration of As at the inlet (S1) also exceeded the probable effect concentration (PEC) (17 mg/kg); however, its concentration at the outlet sampling site (S5) was low and was not detected. The mean concentration of Pb was under the probable effect concentration (PEC) limits (91.13 mg/kg) [35].

The mean concentrations of K, As, Cd, and Cr in this study were significantly lower in the outlet sampling site (S5) than in the inlet sampling site (*P* < 0.05) indicating that the sediment of this wetland is an efficient compartment to retain these metals. However, the mean concentrations of Fe and Pb were significantly higher in the outlet than the inlet sampling sites (*P* < 0.05). This indicated that the sediment in this case is acting as a source rather than a sink for these heavy metals. Once a heavy metal is in a wetland, it may be transported from one compartment to another, e.g., from water to sediments or biota or suspended solids or vice versa. Heavy metals may therefore be removed from polluted wastewater in a wetland and retained in the sediments [36]. When environmental conditions change, sediments may transform from the main sink of heavy metals to sources of them for the overlying waters. According to Prica et al. [37], plant-associated factors, including variation in radial oxygen loss (ROL), pH, microbes, and organic matter, may induce great fluctuations in metal retention and may cause wetlands to become sources for soluble metals instead of the assumed sinks.

The mean metal concentration in the six macrophyte species studied indicated that different species have different tendency of accumulation of metals in their tissue. The mean concentrations of K, Na, Fe, Mn, Cu, Zn, Cr, and As were highest in *Sphaeranthus suaveolens.* Ca and Mg were in highest mean concentration in *Hydrocotyle ranunculoides* and *Echinochloa stagnina*, respectively. The concentrations of Pb and Cd in each species were very low and not detected indicating that these macrophytes are no longer used for retention of these metals or these metals are available in a very limited amount in the environment. The latter is the most probable reason in case of this study. Metal accumulations by macrophytes can be affected by metal concentrations in water and sediments [38]. In this study, the mean concentrations of Pb and Cd in the water column were very low, below detection limit of the instrument (Table 1). Higher mean concentrations of K, Na, Fe, Mn, Cu, Zn, and As were observed in aboveground tissue of *Sphaeranthus suaveolens* and K, Na, Mg, As, and Zn in aboveground tissue of *Hydrocotyle ranunculoides* growing in S2 and S3 (*P* < 0.05). One of the major factors affecting metal accumulation by aquatic plants is duration of exposure [38]. The flat nature of the wetland at S2 and S3 can help the wastewater to stay for long time and enhance macrophytes to absorb and assimilate (accumulate) metals in their tissue. In all the species studied, all the metal concentrations in the aboveground tissue of macrophytes were significantly (*P* < 0.05) lower at the outlet sampling site (S5) if the species is found, indicating that these macrophytes had the ability to absorb metals and act as biofilter for these elements, thereby contributing for the retention of metals in the wetland.

### 4.3. Organic Matter and Metal Removal Efficiency of Yitamot Natural Wetland

In this study, there were evidences that Yitamot wetland system showed high removal efficiency of organic matter: COD (97.46%) (Table 4). The removal success of organic matter could be attributed to microbial activity. Several workers have shown that the roots and structures of macrophytes are able to add an extra filtration component and attachment for microorganisms to further reduce COD ([39–42]). However, though concentrations of all metals in water (except Ca and Mg) decreased at the outlet sampling site (S5), there were no significant removal effects for metal elements like K and Na and heavy metals like Fe, Zn, and As. Mn, Cu, and Cr were the only metals which the wetland showed significant removal, and the other most noticeable feature in this study is the negative percentage for Ca and Mg which represent that the wetland released these metals rather than acting as a sink.

## 5. Conclusion

Measurements of organic matter (COD) and some metal sampling were taken from the three compartments: water, sediment, and plant samples using standard procedures. Findings indicate that the mean concentrations of COD in water showed significant and exponential decrease along the subsequent sampling sites and the mean COD values at the outlet were below the EPA and Ethiopian effluent allowable discharge limits into inland surface waters. The mean concentrations of K, As, Cd, and Cr were significantly lower values in the outlet (S5) than in the inlet (S1) of the sediment samples. All the metal concentrations in the aboveground tissue of macrophytes were significantly lower at the outlet (S5) sample site indicating that these compartments are contributing to the retention of metals and organic matter in the wetland. However, there was no significant removal effect for heavy metals like Fe, Zn, and As. And the mean concentrations of heavy metals As, Cr, Cu, and Fe were higher than the maximum allowable concentration ranges for fisheries and aquatic life set by [24]. The deterioration of water quality in the wetland can cause irreversible loss of the wetland ecosystem in terms of loss of biodiversity and insufficient nutrient and pollutant recovery. It is true that natural wetlands can be used effectively for wastewater treatment with strong monitoring programs and ecological indicators that can be set from other work on nutrient accumulations, flora and fauna targets, etc. These would ensure the functioning of a system and risk mitigation.

## Figures and Tables

**Table 1:** Concentration of COD and metals in water sample of Yitamot wetland (mg/L).

Metal element	Metal concentration in sampling sites (mg/L)						US EPA 1986
	S1	S2	S3	S4	S5	Mean ± SE	
**COD**	207.00^a^	208.25^a^	44.25^m^	37.50^n^	5.25^p^	100.450 ± 990	100.00
K	4.76^b^	3.40^b^	4.20^b^	4.95^b^	4.48^b^	4.360 ± 0.278	
Na	14.91^c^	10.44^c^	10.75^c^	13.88^c^	12.15^c^	12.426 ± 0.722	
Ca	46.26^d^	27.32^d^	50.18^d^	32.50^d^	52.98^d^	41.848 ± 4.840	
Mg	4.20^e^	2.48^e^	4.51^e^	5.96^e^	5.90^e^	4.612 ± 0.540	
Fe	0.81^f^	1.03^f^	23.82^h^	1.00^f^	0.41^f^	5.414 ± 3.071	
Mn	0.11^g^	0.05^g^	0.17^i^	0.02^g^	nd	0.061 ± 0.028	
Cu	1.20^j^	1.08^j^	1.12^j^	1.12^j^	0.59^j^	1.024 ± 0.124	0.007
Zn	1.69^k^	1.13^l^	2.33^l^	1.35^l^	1.47^l^	1.594 ± 0.172	
Pb	nd	nd	nd	nd	nd	nd	0.002
Cr	0.43^q^	0.36^q^	0.42^q^	0.36^q^	0.15^q^	0.343 ± 0.048	0.011
Cd	nd	nd	nd	nd	nd	nd	0.0007
As	0.40^r^	0.41^r^	0.44^r^	0.38^r^	0.38^r^	0.402 ± 0.012	

nd = not detected; SE = standard error of mean. Values followed by the same letters in a raw are not significantly different at *P* < 0.05.

**Table 2:** Concentration of metals in sediment of Yitamot wetland (mg/kg).

Metal	Metal concentration in sampling sites (mg/kg)					
	S1	S2	S3	S4	S5	Mean ± SE
K	123.56	147.21	76.74	25.18	38.27	82.192 ± 16.552
Na	100.99	103.60	58.20	29.15	73.82	73.153 ± 10.163
Ca	59.02	69.34	55.86	24.81	57.53	53.311 ± 5.260
Mg	75.71	40.44	64.50	45.91	28.43	51.000 ± 7.947
Fe	4165.56	4145.12	3738.13	3233.24	5076.79	4071.767 ± 206.186
Mn	75.73	58.80	28.87	16.20	70.98	50.114 ± 8.349
Cu	6.03	6.50	6.90	6.45	5.46	6.267 ± 0.252
Zn	18.80	22.85	17.51	10.92	16.02	17.219 ± 1.557
Pb	0.35	0.21	0.32	0.38	0.87	0.426 ± 0.085
Cr	28.64	28.20	16.73	12.84	12.47	19.776 ± 2.422
Cd	0.07	0.07	0.07	0.04	0.05	0.060 ± 0.005
As	0.75	1.11	0.94	1.53	nd	0.730 ± 0.260

nd = not detected; SE = standard error of mean.

**Table 3:** Mean metal concentration in aboveground plant tissue (mean ± S.D) (mg/kg).

Metal	*Spher. s*	*Hyd. r*	*Cyp. d*	*Ech. s*	*Cyp. p*	*Phr. k*
K	1159.33 ± 323.14	869.31 ± 256.56	243.12 ± 64.96	453.58 ± 55.11	75.87 ± 58.73	119.57 ± 33.44
Na	299.62 ± 102.09	161.07 ± 50.14	33.91 ± 16.57	62.65 ± 19.61	16.46 ± 10.92	4.16 ± 1.41
Ca	111.02 ± 28.33	122.49 ± 24.70	62.91 ± 22.62	87.01 ± 12.31	15.29 ± 11.65	27.05 ± 8.41
Mg	17.56 ± 4.22	20.73 ± 5.13	11.77 ± 4.17	30.33 ± 11.60	2.48 ± 1.84	4.28 ± 1.20
Fe	213.03 ± 96.11	70.93 ± 24.20	45.00 ± 5.19	83.00 ± 13.66	2.84 ± 2.01	2.41 ± 0.69
Mn	7.44 ± 2.62	5.62 ± 1.69	6.47 ± 2.05	7.11 ± 1.07	5.25 ± 3.98	2.91 ± 1.09
Cu	0.85 ± 0.22	0.62 ± 0.13	0.25 ± 0.09	0.68 ± 0.10	0.08 ± 0.06	0.31 ± 0.09
Zn	4.22 ± 1.34	2.45 ± 0.66	1.78 ± 0.94	3.06 ± 0.74	0.30 ± 0.22	0.90 ± 0.31
Pb	0.05 ± 0.01	nd	nd	nd	nd	nd
Cr	0.81 ± 0.38	0.22 ± 0.05	0.14 ± 0.07	0.38 ± 0.08	0.04 ± 0.03	0.14 ± 0.04
Cd	nd	nd	nd	nd	nd	nd
As	0.94 ± 0.35	0.40 ± 0.19	0.15 ± 0.03	0.50 ± 0.25	0.11 ± 0.08	0.29 ± 0.10

nd = not detected; SD = standard deviation.

**Table 4:** Percentage of pollutant removal efficiency using Yitamot wetland.

Pollutant (mg/L)	Inlet	Outlet	Removal efficiency (%)
Mn	0.11	nd	>99.99
COD	207.00	5.25	97.46
Cr	0.43	0.15	64.59
Cu	1.20	0.59	50.48
Fe	0.81	0.41	48.93
Na	14.91	12.15	18.55
Zn	1.69	1.47	12.88
K	4.76	4.48	5.88
As	0.40	0.38	5.56
Ca	46.26	52.98	-14.53
Mg	4.20	5.90	-40.41
Pb	nd	nd	nd
Cd	nd	nd	nd

nd = not detected.

## Data Availability

All data generated or analyzed during this study are included in this published article.
